# Evaluating the effect of assay preparation on the uptake of gold nanoparticles by RAW264.7 cells

**DOI:** 10.1186/s12951-014-0045-5

**Published:** 2014-11-26

**Authors:** Simona Bancos, Katherine M Tyner

**Affiliations:** Center for Drug Evaluation and Research, Food and Drug Administration, Building 51 Room 4159, 10903 New Hampshire Ave., Silver Spring, MD 20993 USA

**Keywords:** Gold nanoparticle, Nanoparticle uptake, *In vitro* assay, Nanoparticle dosimetry, Macrophage

## Abstract

**Background:**

Cell culture conditions can greatly influence the results of nanoparticle (NP) uptake assays. In this study, 10 nm gold nanoparticles (AuNPs) and RAW 264.7 macrophages were used as a model system, while instrumental neutron activation analysis (NAA) was used as the elemental analysis technique to determine AuNP levels produced by the various culturing conditions. Static plate-based and insert-based culture conditions were compared with a dynamic suspension culture to evaluate the conditions’ effect on the rate and extent of AuNP uptake.

**Results:**

The results indicate that a dynamic culturing condition allows for the greatest NP uptake (approximately 3-5 times over the adherent conditions), whereas the plate-based assays have the initial highest rate of NP incorporation.

**Conclusions:**

These data highlight the importance of judiciously choosing the assay conditions prior to evaluating NP uptake.

## Introduction

*In vitro* assays are commonly used during the drug discovery process to provide a rapid assessment of a wide variety of pharmacological endpoints including drug uptake, cell proliferation, cytotoxicity, etc. With the emergence of nanotechnology in medicine, using these *in vitro* assays for drugs containing nanomaterials was a logical continuation of the discovery pathway. However, due to multiple aspects of nanomaterials, including high surface area, enhanced surface activity, and the particulate nature of many drugs incorporating nanomaterials, the direct translation of these *in vitro* assays to nanomaterials has not been straightforward. There have been multiple reports throughout the literature of nanomaterials interfering with cell-based *in vitro* assays, producing false positives, false negatives, or nonsensical data [1,2].

There have been multiple studies evaluating nanoparticle (NP) dosimetry in *in vitro* systems. Teeguarden et al [3] reviewed the many ways different particle dosimetry metrics may impact particle uptake and analysis. In particular, sedimentation through gravitation and agglomeration were highlighted as was the appropriate calculation of delivered NP dose [3]. These considerations are now widespread throughout the field, although some groups have found that there is little effect of sedimentation with some NPs, eliminating many artifactual dosing concerns [4]. In order to demonstrate and/or alleviate the effect of NP sedimentation on apparent NP cellular uptake, several groups have attempted non-conventional culturing techniques. For example, Cho et al. [5] used gold NPs and either traditional plate based assays or inverted assays to monitor cellular uptake of NPs.

There are multiple methods that may be used to monitor NP uptake, each with their unique set of benefits and restrictions. For example, microscopy techniques have been used to determine cellular uptake of nanomaterials. For optical microscopy, sample preparation is often facile, however, NPs must either be labeled or intrinsically fluorescent/luminescent/reflectant, as well as be large enough to be discriminated in the image. Transmission electron microscopy (TEM) can also be used for a confirmation of cellular uptake, but it is rarely used as a direct or quantitative measure of uptake due to the extremely small sample size evaluated. For non-carbon NPs, quantitative assays had been developed to evaluate nanomaterial levels inside the cells such as chemiluminescence measurements, inductively coupled plasma mass spectrometry (ICP-MS), laser desorption/ionization mass spectrometry, and UV-Vis spectrometry [6,7]. Many of these techniques require dilution or digestion of cellular matrix and NP, introducing variability into the measurements, especially for small sample sizes and hard-to-digest materials.

Instrumental neutron activation analysis (NAA) is an elemental analysis technique that can detect gold down to the parts per billion (ppb) level. While not a widespread technique due, in part, to the requirement of a nuclear reactor in order to irradiate the samples, it has some benefits, particularly in the area of sample preparation. The most obvious benefit is the ability to interrogate samples “as is” (i.e. with no digestion or additional sample preparation steps). When interrogating hundreds of samples at a time, this is a non-trivial consideration. In addition, the lack of manipulation may reduce sample variability, due to the reduction of sample processing steps. In this study, NAA is used as the elemental analysis technique to evaluate the uptake of 10 nm AuNPs by the mouse macrophage-like cell line RAW264.7 under different assay conditions. Experimental parameters and culture conditions were varied to determine their impact on the cellular uptake.

## Results and discussion

### NPs characterization

**Figure 1:**
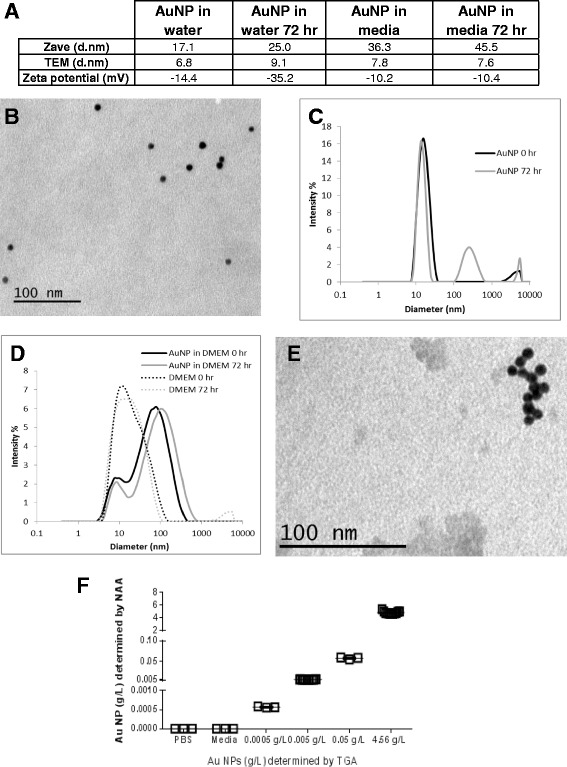
**AuNPs (10 nm) characterization by DLS, TEM and NAA. A**. Summary of size and zeta potential results for AuNPs dispersed in water and media before and after 72 hours incubation at 37°C. **B**. TEM micrograph of 10 nm AuNP dispersed in water. **C**. Representative DLS histogram (intensity weighted) for 10 nm AuNPs before and after incubation at 37°C for 72 hours. **D**. DLS of AuNPs dispersed in media and media control before and after incubation at 37°C for 72 hours. **E**. TEM micrograph of 10 nm AuNPs dispersed in full cell culture media. **F**. Determination of AuNPs concentration by NAA as compared to TGA.

DLS and TEM were used to monitor the size of the AuNPs. Figure 1A contains the summary of the size characterization of the AuNPs dispersed in both media and water before and after incubation at 37°C. In general, NPs dispersed in water were spherical and approximately 10 nm in diameter (Figure 1B), with some agglomeration noted in the intensity weighted DLS histogram as well as the TEM micrographs (Figure 1B-C). This agglomeration translates into a larger overall Zave seen in Figure 1A. Agglomeration of the NPs in water increased over the 72 hour incubation at 37°C as shown by both the increase in Zave and intensity weighted histogram (as indicated by the appearance and increase of additional peaks at larger diameters). Zeta potential decreased during this time period.

To better understand the AuNPs under experimental conditions (full media at 37°C over 72 hours), AuNPs were also dispersed in cell culture media. DLS data is confounded by the presence of serum proteins, which nominally have the same size as the AuNPs (Figure 1D). However, a large peak not present in the media control appears after the addition of the AuNPs. This peak shift and broadening, along with the increase in Zave and lessening of zeta potential is consistent with serum protein opsonization of the AuNPs [8]. Observations with TEM also noted more agglomerates, although this is a qualitative observation (Figure 1E). Further agglomeration was also noted over time in both DLS and TEM. No change was observed in the media control parameters. AuNPs tested below detection levels for endotoxin as determined by *Limulus amoebocytes* lysate (LAL) gel clot assay.

In order to verify AuNPs dosing solution concentrations, culture media containing increasing concentrations of AuNPs (10 nm; 0.0005 g/L- 0.05 g/L) along with AuNPs stock concentration (4.56 g/L as determined by TGA) and controls were prepared and assayed by NAA (Figure 1F). PBS and media without AuNPs were used as negative controls. PBS and control media did not have any detectable levels of AuNPs. For the media dilutions as well as the stock solutions, the expected media concentrations determined by TGA corresponded to the actual concentrations as measured by NAA.

### NAA limits of detection

NAA was used as an elemental analysis technique to eliminate the need for additional sample processing steps. In order to determine the utility of the technique for the study, the limits of detection within the cell line was first determined. NAA detected AuNPs levels in all AuNPs treated macrophage groups. The range was ~0.01 μg (in 0.2 × 10^4^ cells exposed to 0.01 g/L AuNPs) to ~14.5 μg (in 20 × 10^4^ cells exposed to 0.1 g/L). The background levels (cells exposed to media without AuNPs) were 0.001- 0.03 μg. By normalizing these values to cell number, the results indicate that NAA can detect AuNP concentrations in as few as 2000 RAW 264.7 cells. Previous work has shown that the majority of the AuNPs are internalized within the cells and not adhered to the outer membrane [9].

### Time dependent uptake of AuNPs in different cell culture conditions

The time dependent uptake of AuNPs by RAW264.7 cells was evaluated using different culture set-ups. RAW 264.7 cells present an adherent phenotype and are typically cultured in plates in upright conditions. This “basic plate” set-up represents the typical cell assay configuration, where cells are grown in adherent conditions in a multi-well plate, and dosed by media containing the test compound covering the cells. However, in this set-up, there exists the possibility that NPs will settle onto the cell surface which can cause non-representative uptake [3]. To avoid this scenario, a second type of adherent conditions termed “insert” was employed. In this set-up, cells were cultured in a trans-well membrane and covered with media on the apical side. The basal side of the membrane contained the dosing solution (media with AuNPs). The insert set-up allows the RAW 264.7 cells to incorporate AuNPs that are only dispersed within the bulk media, thus avoiding artifacts of uptake due to NP sedimentation on the cell or plate surface.

**Figure 2:**
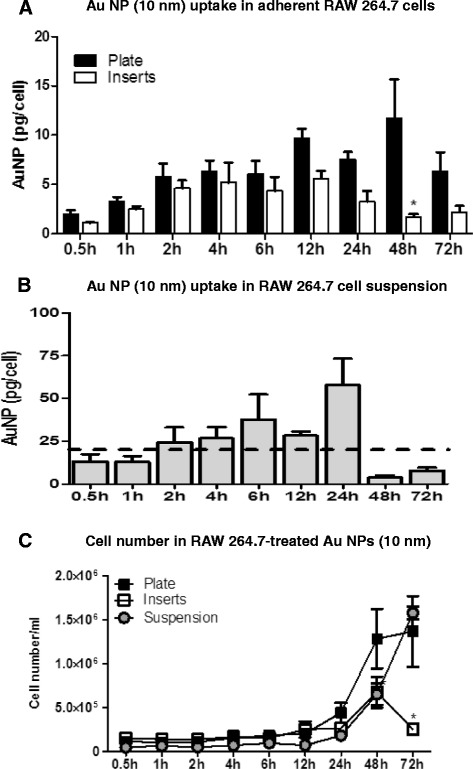
**AuNP (10 nm) uptake in RAW 264.7 cells cultured in adherent (plate and insert) or suspension set-up and their proliferation pattern. A**. AuNP (10 nm) uptake in RAW 264.7 cells show a time-dependent pattern with the highest concentrations determined at 12 h for both traditional and insert set-up. There was a significant difference between AuNPs measured in cells grown in plate versus AuNPs in cells cultured in insert set-up. **B**. AuNP uptake in RAW 264.7 cells cultured in suspension. The peak in AuNP (10 nm) uptake (pg/cell) was detected at 24 h. Except for the 24 h time-point all the time-points were close to the background levels. **C**. RAW 264.7 cells cultured in plates and in suspension proliferate the most when exposed to AuNPs (10 nm) compared to RAW 264.7 cells grown in inserts. Graph shows mean and SEM (N = 6; 2 experiments for Figure 4A and N = 3, 1 experiment for Figure 4B) *, p < 0.05 as determined by two-way ANOVA when comparing traditional to insert set-up.

Figure 2A shows the time course of AuNPs (10 nm) incorporation as monitored by pg AuNP/cell in adherent culture conditions (plate and insert set-up). In general, the uptake profiles are similar with an increase in AuNP concentration per cell over time, which peaks at ~12 hr. After 12 hours, the amount of AuNP per cell levels out and then has decreased by 72 hours. Throughout the time course, AuNP incorporation is slightly higher in the plate set-up versus insert set-up and reaches a statistically significant difference at 48 h. It is important to mention that the level of AuNP was measured by NAA in a cell batch with a known number of cells, after which the value of AuNPs obtained in that batch was divided by the number of cells to obtain AuNP/cell. This calculation, however, does not take in consideration the heterogeneous uptake of RAW267.4 cells, but shows an average for the cell population.

RAW264.7 cells can also be cultured in suspension when using Teflon inserts and gentle agitation to prevent cell adhesion. Figure 2B shows the AuNP uptake of cells under suspension conditions. In this case, the peak AuNP/cell is reached by ~24 hours. The analysis of the uptake, however, is confounded by the assay configuration. In the suspension set-up, the cells are harvested via centrifugation. Due to opsonization and the agglomeration noted in the stability studies, however, many of the AuNPs not incorporated with the cells sediment and form a pellet along with the cell pellet. It is, therefore, difficult to separate the AuNPs in media from the AuNPs incorporated by cells. In this manner, NAA suffers from the same limitation as those in many bulk elemental analysis techniques (such as ICPMS) in that there is no easy way to differentiate between internalized NPs and ones that co-precipitated during the course of the assay. In order to determine the background values (caused by AuNPs in the media sedimenting with the cell pellet), “no cell” controls were performed. These controls were prepared in the same way as the experimental samples, with the exception that cells were not included. NAA of the controls indicates a high background of free AuNPs, which is indicated by the dashed line in Figure 2B. Figure 2B shows the amount of AuNP per cell as compared to the background levels at 24 hours (dashed line). In general, the uptake profile trend is similar to the adherent set-ups with an initial increase in AuNPs/cell, with a decrease after the 24 hour time point.

All tested cell culture set-ups show an initial increase in cell incorporation followed by a decrease by the last time point of 72 hours. In order to understand the uptake profile, the cell proliferation profile was evaluated. Figure 2C shows the number of cells that correspond to each time point for the different set-ups. RAW264.7 cells have a typical doubling time of approximately 12 hours for a standard plate set-up. RAW 264.7 cells exposed to AuNPs (10 nm) proliferated the most when cultured in plate set-up and in suspension. At 48 h and 72 h there are ~2-5 times more cells in plate and suspension versus culture insert. Cells grown on inserts, however, show a reduction in growth at 72 hours, most likely due to cell overgrowth conditions on the insert surface. In adherent conditions (plate and inserts) RAW 264.7 cells reached ~100% confluency at 72 h. It should be noted that the concentration of AuNPs used in this experiment have been previously shown not to cause toxicity in *in vitro* culture conditions [9].

Since, for all the culturing conditions, the amount of AuNPs available is a fixed amount during the time course of the experiment, by 24 hours, there are less AuNPs available per cell simply due to more cells being present in the wells at t =24 hours compared to t =0 hr. Thus, it is possible that the amount of gold per cell decreases over time. In addition, the concentration of AuNPs is further reduced by incorporation into the cells over time, decreasing the amount of free AuNPs available to cells. This dilution effect may be what is responsible for the lower AuNP incorporation into cells at 48 and 72 hours. Such an effect has been analyzed by Summers et al. [10]. Another possibility is the mechanism of exocytosis, which has been demonstrated to occur for AuNPs in macrophage cell lines [11].

In general, cell proliferation was similar between the different culture conditions up to 6 hours, with AuNP incorporation also increasing steadily among the culture conditions up to 4 hours. When examining the rate of uptake over this time period, the plate-based assay had the highest rate of uptake, with a slope of 6.9 (R^2^ 0.90). The insert and suspension uptake conditions had a lower rate of uptake, with slopes of 2.1 (R^2^ 0.96) and 2.5 (R^2^ 0.98), respectively. The enhanced uptake of the plate-based assay compared to the insert may be explained by the culturing conditions, where the cells cultured in the standard plate assay encounter particles both dispersed throughout the media, as well as any settled agglomerates. In contrast, the cells cultured on the inserts only have access to the AuNPs that remain dispersed in the media. Cho et al. [5] using an inverted set-up demonstrated that cells cultured in an inverted set-up incorporated less AuNPs versus cells cultured in an upright set-up. The difference between the set-ups was least apparent for their smallest AuNPs tested (15 nm). Although the suspension assay conditions have a similar rate of AuNP uptake as the insert set-up, the actual amount of AuNP incorporated per cell is much higher than either the plate or insert-based assays, even when accounting for the baseline AuNPs. Again, culture conditions may inform these results. For the suspension cells, the culturing conditions are not static, with constant movement of the cells and AuNPs (both single and agglomerated) dispersed within the media. These dynamic conditions provide additional interactions between the cells and AuNPs, allowing for enhanced AuNP uptake.

One of the drawbacks of adherent/plate assays is that the exact cell number at the time of experiment can only be approximated (due to the cell proliferation that is occurring during the plate incubation time, e.g. “plates were incubated overnight prior to treatments). The cell numbers can be known exactly when plating, and can be determined after the assay via cell counts, but the number of cells at the time of assay dosing is an approximation. This is another advantage of using cells in suspension. The cell number in our “adherent/plate set-up” conditions were determined in each experiment at the time of plating and at the end of the assay.

### AuNPs distribution and recovery in culture conditions *in vitro*

**Figure 3:**
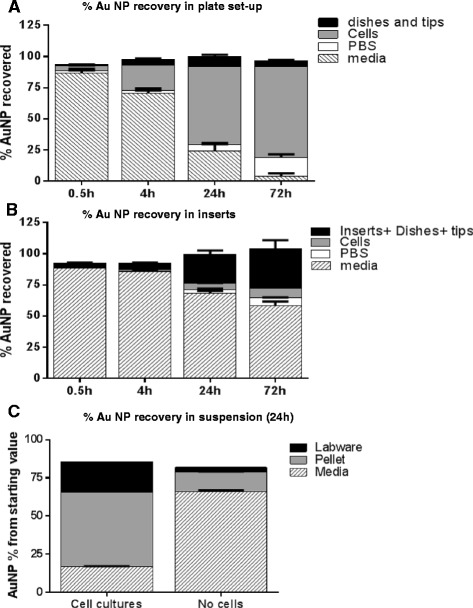
**AuNPs recovery in RAW 264.7 cell cultures.** There is a ~100% recovery of AuNPs in plate **(A)**, insert **(B)** and suspension **(C)** set-up. Graph shows mean and SEM (N = 6; 2 independent experiments for A and B and N = 3 for C).

NPs can adhere to not only external cell membranes, but to the tissue culture plates, pippettor surfaces, etc., reducing the actual dosing concentration of the NPs [12]. In order to have a better understanding of the dosing conditions of the AuNPs within the cell culture set-up, a mass balance study was undertaken for all cell culture conditions. At each time point for the adherent set-ups (and at 24 hours for the suspension set up), media was removed from the cell monolayer, placed in NAA vials, and allowed to dry. PBS used to rinse the cell monolayers was also collected in a separate NAA vial, as was the cell monolayer itself (reserving a small aliquot to use for cell counting). All labware that the AuNPs had contact with (pipettor tips, cell scrapers, culture plate dishes, Teflon inserts) were collected and pooled into a final NAA vial. Figure 3 represents the results of each of the conditions tested in % AuNP recovered. Since in this experiment the aim was to obtain a mass balance (AuNP distribution in cells and culture media throughout the incubation time), AuNPs levels were determined in the harvested cell population and are shown as percentage of the starting AuNPs (0.005 g/L) rather than AuNPs/cell.

For the adherent set-ups (Figure 3A and B), recovery was close to 100% and no less than 90% for any of the time points evaluated. Figure 3A shows the recovery profile for the plate-based set up. At the beginning of the experiment, the majority of the AuNPs are in the media, and a small fraction of the AuNPs within the cells. As the incubation time increases, the fraction of AuNPs within the media decreases with a corresponding increase of the amount of AuNP in the cells, in agreement with the uptake of the AuNPs by the macrophages over time. The amount of AuNPs measured in the PBS rinse also increases over time, and could be a result of dead or dying cells that had incorporated AuNPs and were removed from the cell layer upon rinsing, or a result of an exocytosis mechanism. Only a small fraction of the AuNPs remained adhered to the labware.

As anticipated with the culture inserts set-up, the majority of AuNPs remain in the media portion of the set-up throughout the incubation time, with a minor fraction (up to 14%) measured in the cells (Figure 3B). Towards the end of culture period, the amount of AuNP detected in PBS washes increased to ~15-20% again, likely due to cell death and detachment and/or exocytosis. The amount of AuNPs recovered in the labware increases over time, with the largest percentage found at 72 hours, possibly indicating adhesion/interaction of the AuNPs with the insert membrane over time.

Figure 3C shows the mass balance for the 24 hour time point. Due to the s cost considerations of the Teflon inserts, only the 24 hour time point was evaluated. As there was a high AuNP baseline (as noted above), a media control was also performed for this measurement and is shown in the right side of the figure. At 24 hours, the majority of AuNPs are associated with the cells, with only a small fraction remaining with the media. This result, however, is cofounded by free AuNPs pelleting with the cells. Approximately 13% of the control media pellet contained AuNPs that is not incorporated by the cells. A large portion of the AuNPs also appears with the labware. This may be due to the multiple transfer steps that this set-up requires. These multiple transfers plus not measuring the Petri dishes where the inserts were housed (along with any media drips that occurred during the incubation) may account for the lower recovery of the AuNPs.

The mass balance distribution highlights the differences between the different adherent culture conditions. For the plate based conditions, there is strong correlation between the AuNPs concentration in the media (decreasing over time) and the incorporation of AuNPs within the cell monolayer (increasing over time). Whereas this correlation is also observed for the insert conditions, it is not as robust a response, and correlates with the rate of uptake that was observed in Figure 2, where the cells in plate-based conditions have more access to the AuNPs. Suspension conditions also show a large incorporation of AuNPs within the cells at 24 hours, which also agrees with the rate of uptake studies, where the dynamic culture conditions bring the cells and NPs into contact more frequently.

### AuNP settling studies

**Figure 4:**
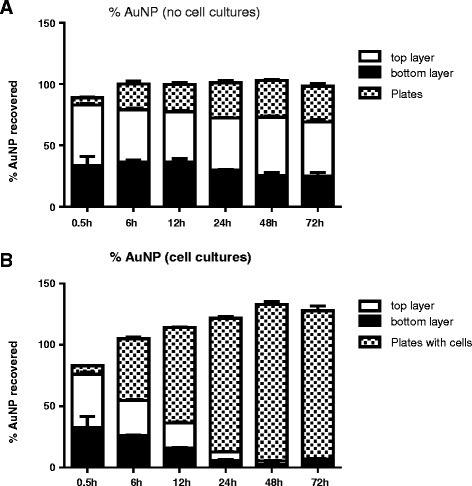
**Settling studies for plate-based set-up with (A) no cells present and (B) cells present.** No significant difference found between time points or between the top and bottom media layers. P < 0.05 two-way ANOVA.

Based upon the stability studies conducted in media (Figure 1), as well as common belief in the literature, it was assumed that the AuNPs would over time agglomerate and settle to the bottom of a plate-based set-up in significant quantities [13]. To test this hypothesis, a settling experiment was conducted where the plate-based set-up was evaluated for the amount of AuNPs in the top aliquot of media, the bottom layer of media, and settled onto the plate. Conditions were run both with and without cells (Figure 4). Figure 4A shows the mass balance results for the plate-set-up without cells. For this experiment, approximately the same amount of AuNPs is found in the top media layer, bottom media layer and on the culture plates throughout the 72 hours of incubation. No statistical difference was found between the top and bottom media layers. For the plates that contained cells (Figure 4B), the amount of AuNPs within the media decreases over the time course of the experiment, while the percentage of AuNPs associated with the plates increases. However, due to the manipulation steps (plates transfer from the incubator to the sterile hood, pipetting, etc) it is difficult to accurately collect the top and bottom layers. Therefore, as shown in Figure 4B, some errors due to the handling of the culture plates occurred. Nevertheless, recovery of AuNPs in the plates containing cells is in line with the uptake experiments presented in Figures 2 and 3, with the cells layer incorporating most of the AuNPs from the media by 72 hours. Again, there was no difference in the gold concentrations in the different media layers.

This lack of significant settling is consistent with the experimental results demonstrated with Cho et al [5], who determined that the uptake of uncoated 15 nm AuNPs was essentially the same in inverted versus plate-based set-ups. Based upon their results, the authors hypothesized that diffusion of NPs within the cell culture media was the main mode of transport (as opposed to sedimentation) for these smaller NPs. Indeed our own DLS stability study, while indicating agglomeration over time, did not indicate significant settling, with no visible settling and no significant decrease of the count rate over the course of the experiment. The lack of significant sedimentation, however, confounds the analysis of the greater cellular uptake of AuNPs in the plate-based setting (compared to the insert culture conditions). Several factors could contribute to this observation. The first is that the membrane insert is impeding free diffusion of the AuNPs to the cell monolayer, thus artificially lowering the AuNP dose. Another possibility is that the control sedimentation conditions used in Figure 4A do not account for the extracellular matrix. This matrix could cause the AuNPs to agglomerate and stick near the macrophages, thus allowing for the observed increased uptake.

### Basic culture conditions impact cellular uptake

**Figure 5:**
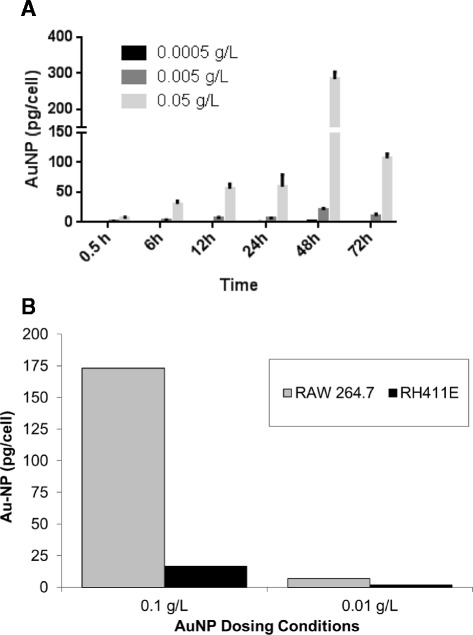
**NP dose and type of cell line impacts the amount of AuNP uptake. (A)** Uptake of AuNPs in RAW264.7 cells when exposed to increasing concentrations of AuNPs. **(B)** AuNP uptake in pg/cell for a hepatocyte cell line (rH4IIE) versus a macrophage cell line (RAW264.7).

Finally, other aspects of the assay set up may greatly influence the uptake of NPs by macrophages. Figure 5A shows the amount of AuNPs taken up by cells in a plate-based set-up and exposed to increasing amounts of gold. Not surprisingly, increasing the amount of AuNPs within the media increases not only the amount of NPs internalized in the cell, but the rate of uptake as well, with the higher AuNP concentrations showing a higher rate of uptake over the first 4 hours (0.0005 g/L, 1.4, R^2^ 0.92; 0.005 g/L, 6.9, R^2^ 0.90; 0.05 g/L, 78.0, R^2^ 0.96). The starting cell density (cells/ NP), on the other hand, did not significantly affect the cellular uptake profile, with the exception of when cells overgrew and died (data not shown).

To better understand NP-induced cytotoxicity *in vitro* several laboratories have developed platforms in which several cell types are exposed to different NPs and their cytotoxicity is evaluated using a variety of techniques and assays. It was shown that 3 T3 fibroblasts are more resistant to NPs (TiO_2_ NPs, SiO_2_ NPs, MWCNT) while RAW 264.7 cells are more sensitive [14]. Using a panel of 10 different cell lines and 23 different NPs it was shown that there were different toxicity responses in the cells analyzed [15]. While this is a thorough approach, a clear conclusion cannot be drawn unless there is a quantitative determination of NPs level in every cell type. The difference in cytotoxcity responses among cell types could be caused by the incorporation of higher NPs levels in some of the cells studied. Our data show that RAW 264.7 cells incorporate more AuNPs versus rat hepatocytes (Figure 5B). This result is not overly surprising given that RAW264.7 cells are derived from a specialized phagocytyic cell line that has been noted for rapid uptake of NP [16]. The rH4IIR hepatocyte cell line, in contrast, has less phagocytic capabilities. The phagocyotic macrophages have almost triple the amount of AuNPs in them after 24 hours as compared to the hepatic cells. These data underscore the importance of using not only the correct assay conditions, but the cells most relevant to the desired endpoint (e.g. if studying toxicity for a possibly hepatotoxicant, a hepatocyte might be more relevant than a phagocytic cell).

## Conclusions

While there is overwhelming information regarding the factors that influence NP uptake *in vitro* (physical and chemical characteristics, dosimetry, cell lines used, etc.) there are few communications related to the actual amount of NPs incorporated inside the cells. This study looked at the effect of different plating conditions on the uptake of AuNPs. While this study only evaluates the uptake in three assay conditions, the techniques and evaluation could be applied toward other in vitro models, including flow-through and 3 dimensional models. The suspension culture, while exhibiting some interference with agglomerated AuNPs showed more uptake than the static systems and has the potential to be more representative *in vivo* (where sedimentation of the NPs onto tissues is less likely to occur). In contrast, the insert culture has the least AuNP uptake but mitigates settling effects as well as the pelleting issues observed for the suspension system. While none of the set-ups employed (plate, inserts and cell suspension) are ideal, the current study draws attention to the variability in NPs incorporation that can be induced by assay set-up as well as the necessity to rationally choose the most appropriate assay conditions.

## Methods

### Reagents

RAW 264.7 cells and DMEM culture media were purchased from ATCC; FBS, Penicillin/Streptomycin (p/s), PBS, propidium iodide (PI) were purchased from Invitrogen. AuNPs (10 nm) were purchased from Structure Probe Inc.

### NPs characterization

AuNPs were concentrated as previously described [8]. Concentration was determined by TGA and later confirmed by NAA. For size and stability analysis, AuNPs were diluted in water or media with 10% FBS and 1% penicillin/streptomycin. Full media without AuNPs were run as a control. AuNPs were dispersed into the water or media at a concentration of 0.005 g/L and then aliquoted into individual zeta cuvettes (Malvern) and stored at 37°C. At various time points, cuvettes were removed and size via dynamic light scattering (DLS) and zeta potential were measured on a Malvern Zetasizer. Parameters were adjusted for viscosity and refractive index for the media and water. Measurement position and attenuator were standardized across all runs. Three measurements of 12 runs each were taken for each time point after an incubation time of 120 sec. For zeta potential measurements, 50 runs were performed. Z average, intensity weighted histograms, and zeta potential were recorded. Lack of endotoxin contamination was confirmed by LAL gel clot formation assay. For TEM analysis an aliquot of AuNPs was triple dripped onto a holey carbon coated copper grid (quantfoil, EMS, PA USA) and allowed to air dry. Grids were analyzed on a JEOL 1400 TEM at 80 kV. At least 5 images were taken for each sample with 36-96 particles counted.

Dosing solutions were prepared based upon AuNPs concentrations determined by TGA. To compare the AuNPs concentration in media as measured by TGA and NAA, an aliquot of media containing AuNPs or the stock solution was placed into an NAA vial and allowed to dry. NAA vials were analyzed within the main cell experiments (see details below). N = 12 for 0.005 g/L and stock concentrations; N = 3 for rest of dilutions.

### RAW 264.7 cell culture conditions

RAW 264.7 cells were purchased from ATCC, propagated and aliquots stored in liquid nitrogen. Cells were maintained as adherent cell cultures and passaged 3-25 times after which a new frozen aliquot was used. Cell treatments were performed in adherent or cell suspension conditions. Cell density was 10^5^ cells/ml at time of AuNP treatment. Cell suspensions were exposed to AuNPs at the time of plating. In adherent cultures, cells were plated at a density of 0.8 × 10^4^ cells/cm^2^ in either 12-well plates (3.8 cm^2^ surface area) or tissues culture Transwell inserts (0.9 cm^2^). For adherent cultures, cells were allowed to propagate for 48 h (at which point they reached a density of ~ 10^5^ cells/well or insert) and were exposed to AuNPs. Cells were harvested and processed depending on experiment requirements (described below).

### Cellular uptake and mass balance studies

For adherent set-ups, RAW 264.7 cells cultured in 12 well multi-well plates were exposed to AuNPs (0.005 g/L) for varying time points, after which supernatants were harvested, cells washed with PBS, trypsinized, counted and transferred into NAA vials. 12 well plates were sectioned to isolate the individual wells for analysis. With the exception of the cells that were used to determine cell count, all cells were used for NAA. RAW 264.7 cells grown in trans-well culture inserts were exposed to theAuNPs only in the basal side of the membrane (media only was added to the top of inserts). When mass balance experiments were performed, cells, culture media, PBS washes, and the labware that came in contact with the AuNPs (pipettor tips, culture inserts and culture wells) were collected, transferred into individual NAA vials and measured for AuNP levels. Conditions were run in triplicate on two separate days for an N = 6.

For suspension cells, RAW 264.7 cells were plated in 60 mm Petri plates containing Teflon inserts in 5 mL medial containing 0.005 g/L AuNPs. Cells were placed on a shaker table within the incubator during the experiment. At variable time points, cells were removed from the inserts with a cell scraper and transferred to centrifuge tubes. Cells were centrifuge at 1500 rpm (~500 g) for 10 minutes at room temperature. The supernatant was saved in a NAA vial and the cell pellet resuspended in 500 μL PBS. Cells were counted and transferred to an NAA vial for analysis. For mass balance experiments, the experimental set up separated cells, culture media, PBS washes, and labware that came in contact with the AuNPs (pipettor tips, cell scraper, centrifuge tube, and Teflon inserts). Conditions were run in triplicate on two separate days for an N = 6.

### NAA limits of detection

RAW 264.7 cells were plated at a density of 0.2 × 10^4^ – 20 × 10^4^ cells/measurement. Cells were exposed to media with no AuNPs or media containing 0.01 g/L or 0.1 g/L AuNPs for 24 h and processed as above.

### Settling studies

RAW 264.7 cells cultured in 12 well multi-well plates (as noted above) were exposed to AuNPs (0.005 g/L) for varying time points. 400 μL of media was carefully aspirated from the top of the well to avoid dispersing AuNPs and transferred to an NAA vial. The remaining media was then transferred to a NAA vial. Plates were then sectioned to isolate the individual wells and the wells were placed in an NAA vial. For the media control, the above procedure was repeated with the exception that no cells were cultured in the plates. The experiment was run in triplicate with N =3.

### NAA

Sample vials were allowed to dry prior to capping the vials to prevent leakage during sample transfer and analysis. For the 12 well plates, plates were dried in a hood prior to sectioning. NAA analysis was performed at Becquerel Laboratory (Ontario, Canada) using their standard operation procedure SOP (BQ-NAA-4, Elemental Analysis via INAA) through a contract with Elemental Analysis Inc (Kentucky, USA). Gold values were reported as total gold in μg/vial.

Statistical analyses were performed using two-way ANOVA followed by Bonferroni’s multiple comparison tests.
